# Supplementary material for: Efficient base editing for multiple genes and loci in pigs using base editors
Source: Nat Commun. 2019 Jun 28;10:2852. doi: 10.1038/s41467-019-10421-8 (PMC6599043; doi:10.1038/s41467-019-10421-8)
Supplement: Supplementary file 1 — Supplementary Information [file 41467_2019_10421_MOESM1_ESM.pdf]

Efficient base editing for multiple genes/loci in pigs using base editors

Supplementary Information

Xie *et al*. 2019

Supplementary Fig. 1

a

DMD	5′-TTTTTTTATCTTTCAGGTGCTTCAGGAAGACCTAGAGCAGGAACAAGTCAGGGTCAATTC-3′	**Wild-type**	
***DTL*-E4:**	5′-TTTTTTTATCTTTCAGGTGCTT**T**AGGAAGACCTAGAGCAGGAACAAGTCAGGGTCAATTC-3′	(13/20)	(Q493STOP)
***DTL*-E11:**	5′-TTTTTTTATCTTTCAGGTGCTT**T**AGGAAGACCTAGAGCAGGAACAAGTCAGGGTCAATTC-3′	(6/20)	(Q493STOP)
TYR	5′-GAGGTGATGGGAGTCCCTGTGGCCAGCTCTCAGGCAGGGGTTCCTGTCAGGACATCATTC-3′	**Wild-type**	
***DTL*-E4:**	5′-GAGGTGATGGGAGTCCCTGTGG**TT**AGCTCTCAGGCAGGGGTTCCTGTCAGGACATCATTC-3′	(13/20)	(G27G, Q28STOP)
	5′-GAGGTGATGGGAGTCCCTGTGG**A**CAGCTCT**T**AGGCAGGGGTTCCTGTCAGGACATCATTC-3′	(7/20)	(G27G, S30L)
***DTL*-E11:**	5′-GAGGTGATGGGAGTCCCTGTGG**TT**AGCTCTCAGGCAGGGGTTCCTGTCAGGACATCATTC-3′	(10/20)	(G27G, Q28STOP)
LMNA	5′-AGCAGCTCGGGAGCCCAGGTGGGCGGATCCATCTCCTCTGGCTCCTCCGCCTCCAGTGTC-3′	**Wild-type**	
***DTL*-E4:**	5′-AGCAGCTCGGGAGCCCAGGTGGG**T**GGATCCATCTCCTCTGGCTCCTCCGCCTCCAGTGTC-3′	(4/20)	(G608G)
	5′-AGCAGCTCGGGAGCCCAGGTGGG**T**GGAT**T**CATCTCCTCTGGCTCCTCCGCCTCCAGTGTC-3′	(4/20)	(G608G, S610F)
***DTL*-E11:**	5′-AGCAGCTCGGGAGCCCAGGTGGG**T**GGATCCATCTCCTCTGGCTCCTCCGCCTCCAGTGTC-3′	(20/20)	(G608G)

b

RAG1	5′-AGGGGAAACCCTCGCTGGAGCAATCTCCAGCAGTCCTGGACAAGCCTGGTGGTCAGAAG-3′	**Wild-type**	
***R12I*-E3:**	5′-AGGGGAAACCCTCGCTGGAG**T**AATCTCCAGCAGTCCTGGACAAGCCTGGTGGTCAGAAG-3′	(5/20)	(Q61STOP)
***R12I*-E17:**	5′-AGGGGAAACCCTCGCTGGAG**T**AATCTCCAGCAGTCCTGGACAAGCCTGGTGGTCAGAAG-3′	(8/20)	(Q61STOP)
	5′-AGGGGAAACCCTCGCTGGAG**T**AAT**T**TCCAGCAGTCCTGGACAAGCCTGGTGGTCAGAAG-3′	(3/20)	(Q61STOP,S62F)
RAG2	5′-CCCACTGTCAGAAAATGTCACTACAGATGATAACAGTTGGTAATAACATGGCCTTAATT-3′	**Wild-type**	
***R12I*-E3:**	5′-CCCACTGTCAGAAAATGT**T**ACTA**T**AGATGATAACAGTTGGTAATAACATGGCCTTAATT-3′	(3/20)	(S2L,Q4STOP)
***R12I*-E17:**	5′-CCCACTGTCAGAAAATGTCACTA**T**AGATGATAACAGTTGGTAATAACATGGCCTTAATT-3′	(10/20)	(Q4STOP)
	5′-CCCACTGTCAGAAAATGT**T**A**T**TA**T**AGATGATAACAGTTGGTAATAACATGGCCTTAATT-3′	(10/20)	(S2L,L3L,Q4STOP)
IL2RG	5′-CTACCAAACATTTGTTGTCCAGCTCCAGGACCCACGGGAACCCAGGAGGCAGGACCCAC-3′	**Wild-type**	
***R12I*-E4:**	5′-CTACCAAACATTTGTTGT**TT**AGCT**TT**AGGACCCACGGGAACCCAGGAGGCAGGACCCAC-3′	(4/20)	(V154V,Q155STOP,L156L,Q157STOP)
***R12I*-E17:**	5′-CTACCAAACATTTGTTGT**TT**AGCTCCAGGACCCACGGGAACCCAGGAGGCAGGACCCAC-3′	(15/20)	(V154V,Q155STOP)
	5′-CTACCAAACATTTGTTGT**TT**AG**T**TCCAGGACCCACGGGAACCCAGGAGGCAGGACCCAC-3′	(5/20)	(V154V,Q155STOP,C156F)

Supplementary Fig. 1 Simultaneous base editing of *DMD*, *TYR* and *LMNA*, or *RAG1*, *RAG2* and *IL2RG* in porcine embryos. a and **b** T-cloning and Sanger sequencing to detect base editing of DTL-E4, E17 (**a**), and R12I-E3, E17 (**b**). The sequences of sgRNAs are marked in yellow and the PAM sites sequences are labeled in green. The targeted sequence is underlined.

Supplementary Fig. 2

a

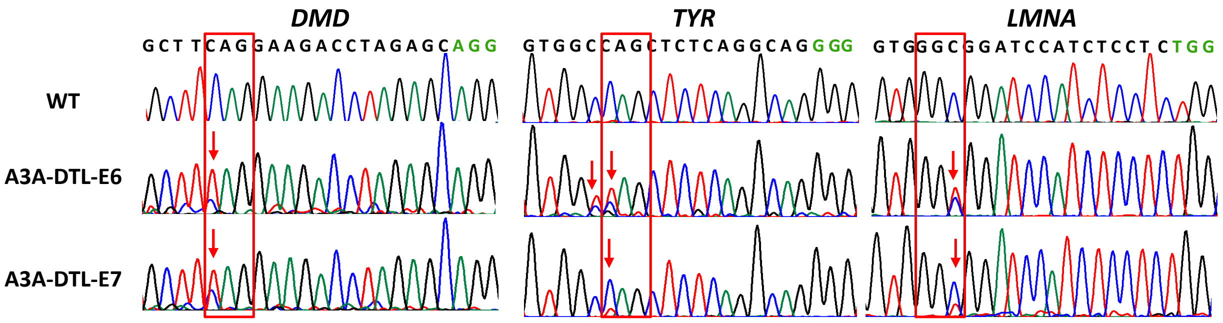

b

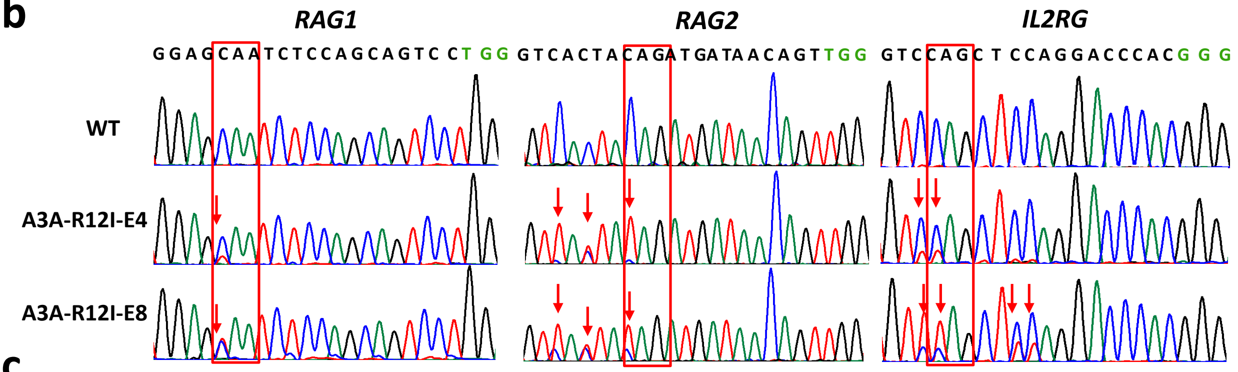

c

DMD 5′-TTTTTTTATCTTTCAGGTGCTTCAGGAAGACCTAGAGCAGGAACAAGTCAGGGTCAATTC-3′ **Wild-type**

***DTL-E6*:** 5′-TTTTTTTATCTTTCAGGTGCTT**T**AGGAAGACCTAGAGCAGGAACAAGTCAGGGTCAATTC-3′ (14/20) (Q493STOP)

***DTL-E7*:** 5′-TTTTTTTATCTTTCAGGTGCTT**T**AGGAAGACCTAGAGCAGGAACAAGTCAGGGTCAATTC-3′ (12/20) (Q493STOP)

TYR 5′-GAGGTGATGGGAGTCCCTGTGGCCAGCTCTCAGGCAGGGGTTCCTGTCAGGACATCATTC-3′ **Wild-type**

***DTL-E6*:** 5′-GAGGTGATGGGAGTCCCTGTGG**TT**AGCTCTCAGGCAGGGGTTCCTGTCAGGACATCATTC-3′ (11/20)(G27G,Q28STOP)

5′-GAGGTGATGGGAGTCCCTGTGG**CG**AGCTCT**C**AGGCAGGGGTTCCTGTCAGGACATCATTC-3′ (1/20) (G27G,S30L)

***DTL-E7*:** 5′-GAGGTGATGGGAGTCCCTGTGGC**T**AGCTCTCAGGCAGGGGTTCCTGTCAGGACATCATTC-3′ (2/20) (G27G,Q28STOP)

LMNA 5′-AGCAGCTCGGGAGCCCAGGTGGGCGGATCCATCTCCTCTGGCTCCTCCGCCTCCAGTGTC-3′ **Wild-type**

***DTL-E6*:** 5′-AGCAGCTCGGGAGCCCAGGTGGG**T**GGATCCATCTCCTCTGGCTCCTCCGCCTCCAGTGTC-3′ (11/20)(G608G)

***DTL-E7*:** 5′-AGCAGCTCGGGAGCCCAGGTGGG**T**GGATCCATCTCCTCTGGCTCCTCCGCCTCCAGTGTC-3′ (4/20) (G608G)

d

RAG1 5′-AGGGGAAACCCTCGCTGGAGCAATCTCCAGCAGTCCTGGACAAGCCTGGTGGTCAGAAG-3′ **Wild-type**

***R12I-E4*:** 5′-AGGGGAAACCCTCGCTGGAG**T**AATCTCCAGCAGTCCTGGACAAGCCTGGTGGTCAGAAG-3′ (3/20) (Q61STOP)

***R12I-E8*:** 5′-AGGGGAAACCCTCGCTGGAG**T**AATCTCCAGCAGTCCTGGACAAGCCTGGTGGTCAGAAG-3′ (10/20) (Q61STOP)

RAG2 5′-CCCACTGTCAGAAAATGTCACTACAGATGATAACAGTTGGTAATAACATGGCCTTAATT-3′ **Wild-type**

***R12I-E4*:** 5′-CCCACTGTCAGAAAATGT**T**AT**T**A**T**AGATGATAACAGTTGGTAATAACATGGCCTTAATT-3′ (10/20)(S2L,L3L,Q4STOP)

5′-CCCACTGTCAGAAAATGT**T**ACTA**T**AGATGATAACAGTTGGTAATAACATGGCCTTAATT-3′ (9/20) (S2L,Q4STOP)

***R12I-E8*:** 5′-CCCACTGTCAGAAAATGT**T**AT**T**A**T**AGATGATAACAGTTGGTAATAACATGGCCTTAATT-3′ (10/20)(S2L,L3L,Q4STOP)

5′-CCCACTGTCAGAAAATGT**T**ACTA**T**AGATGATAACAGTTGGTAATAACATGGCCTTAATT-3′ (7/20) (S2L,Q4STOP)

IL2RG 5′-CTACCAAACATTTGTTGTCCAGCTCCAGGACCCACGGGAACCCAGGAGGCAGGACCCAC-3′ **Wild-type**

***R12I-E4*:** 5′-CTACCAAACATTTGTTGT**TT**AGCTCCAGGACCCACGGGAACCCAGGAGGCAGGACCCAC-3′ (4/20) (V154V,Q155STOP)

***R12I-E8*:** 5′-CTACCAAACATTTGTTGT**TT**AGCTCCAGGACCCACGGGAACCCAGGAGGCAGGACCCAC-3′ (9/20) (V154V,Q155STOP)

5′-CTACCAAACATTTGTTGT**TT**AGCT**TT**AGGACCCACGGGAACCCAGGAGGCAGGACCCAC-3′ (6/20) (V154V,Q155STOP,L156L,Q157STOP)

e

No. of embryos injected	No. of blastocysts (%)	No. of blastocysts detected	No. of blastocysts with single gene base editing (%)			No. of blastocysts with two genes base editing (%)			No. of blastocysts with three genes base editing (%)
			DMD	*TYR*	*LMNA*	*DMD* and *TYR*	*TYR* and *LMNA*	*DMD* and *LMNA*	*DMD*, *TYR* and *LMNA*
100	20 (24)	10	0	0	0	1 (10.0)	0	2 (20.0)	5 (50.0)
			RAG1	*RAG2*	*IL2RG*	*RAG1* and *RAG2*	*RAG1* and *IL2RG*	*RAG2* and *IL2RG*	*RAG1*, *RAG2* and *IL2RG*
100	25 (25)	12	1 (8.3%)	0	1 (8.3%)	1 (8.3%)	0	1 (8.3%)	5 (41.7)

Supplementary Fig. 2 hA3A-BE3-mediated multiple genes base editing in porcine embryos. a and **b** Sanger sequencing results of the target sites in A3A-DTL-E6, E7 (**a**) and A3A-R12I-E4, E6 (**b**). Target base editing was labeled with red arrows. **c** and **d** T-cloning and Sanger sequencing of base editing of A3A-DTL-E6, E7 (**c**) and A3A-R12I-E4, E6 (**d**). PAM sites are highlighted in green; target sequences are red. **e** Summary of multiple sites base editing by hA3A-BE3 in porcine embryos

Supplementary Fig. 3

a

Blastocysts	C to T	C to G	WT	Ratio of base editing
pol-E1	57.8%	29.5%	12.7	87.3%
pol-E2	70.8%	20.5%	8.7%	91.3%
pol-E3	78.9%	21%	0	100%
pol-E4	35.3%	11.8%	52.9%	47.1%
pol-E5	72.9%	13.5%	13.6%	86.4%
pol-E6	71.0%	18.0%	11.0%	89.0%
pol-E7	42.5%	25.6%	31.9%	68.1%
pol-E8	40.0%	22.5%	37.5%	62.5%
pol-E9	54.2%	24.5%	21.3%	78.7%
pol-E10	54.4%	32.6%	13.0%	87.0%
pol-E11	48.2%	30.2%	22.6%	78.4%
pol-E12	44.7%	20.9%	34.4%	65.6%
pol-E13	29.5%	46.8%	23.7%	76.3%
pol-E14	46.8%	26.3%	26.9%	73.1%
pol-E15	55.2%	25.9%	18.9%	81.1%
pol-E16	58.0%	17.1%	24.9%	75.1%
pol-E17	63.9%	21.3%	14.8%	85.2%
pol-E18	56.5%	25.3%	18.2%	81.8%
pol-E19	65.5%	19.8%	14.7%	85.3%
pol-E20	61.7%	16.5%	21.8%	78.2%

b

No. of embryos injected	No. of blastocysts (%)	No. of blastocysts detected	No. of blastocysts with *pol* gene base editing (%)
100	20 (20%)	20	20 (100%)

Supplementary Fig. 3 Base editing of the *pol* gene in porcine embryos. a and **b** Detailed information concerning base editing efficiency of each injected porcine embryo.

Supplementary Fig. 4

a

***DTL* mutant cell colonies**

DMD	5'-TTTTTTTATCTTTCAGGTGCTTCAGGAAGACCTAGAGCAGGAACAAGTCAGGGTCAATTC-3'	**Wild-type**
***DTL*-C1:**	5'-TTTTTTTATCTTTCAGGTGCTTTAGGAAGACCTAGAGCAGGAACAAGTCAGGGTCAATTC-3'	(Q493STOP)
***DTL*-C23:**	5'-TTTTTTTATCTTTCAGGTGCTTTAGGAAGACCTAGAGCAGGAACAAGTCAGGGTCAATTC-3'	(Q493STOP)
***DTL*-C37:**	5'-TTTTTTTATCTTTCAGGTGCTTCAGGAAGACCTAGAGCAGGAACAAGTCAGGGTCAATTC-3'	
	5'-TTTTTTTATCTTTCAGGTGCTTTAGGAAGACCTAGAGCAGGAACAAGTCAGGGTCAATTC-3'	(Q493STOP)
TYR	5'-GAGGTGATGGGAGTCCCTGTGGCCAGCTCTCAGGCAGGGGTTCCTGTCAGGACATCATTC-3'	**Wild-type**
***DTL*-C1:**	5'-GAGGTGATGGGAGTCCCTGTGGTTAGCTCTCAGGCAGGGGTTCCTGTCAGGACATCATTC-3'	(G27G, Q28STOP)
***DTL*-C23:**	5'-GAGGTGATGGGAGTCCCTGTGGCCAGCTCTCAGGCAGGGGTTCCTGTCAGGACATCATTC-3'	
	5'-GAGGTGATGGGAGTCCCTGTGGCTAGCTCTCAGGCAGGGGTTCCTGTCAGGACATCATTC-3'	(Q28STOP)
***DTL*-C37:**	5'-GAGGTGATGGGAGTCCCTGTGGCCAGCTCTCAGGCAGGGGTTCCTGTCAGGACATCATTC-3'	
	5'-GAGGTGATGGGAGTCCCTGTGGTTAGCTCTCAGGCAGGGGTTCCTGTCAGGACATCATTC-3'	(G27G, Q28STOP)
LMNA	5'-AGCAGCTCGGGAGCCCAGGTGGGCGGATCCATCTCCTCTGGCTCCTCCGCCTCCAGTGTC-3'	**Wild-type**
***DTL*-C1:**	5'-AGCAGCTCGGGAGCCCAGGTGGGCGGATCCATCTCCTCTGGCTCCTCCGCCTCCAGTGTC-3'	
	5'-AGCAGCTCGGGAGCCCAGGTGGGTGGATCCATCTCCTCTGGCTCCTCCGCCTCCAGTGTC-3'	(G609G)
***DTL*-C23:**	5'-AGCAGCTCGGGAGCCCAGGTGGGCGGATCCATCTCCTCTGGCTCCTCCGCCTCCAGTGTC-3'	
	5'-AGCAGCTCGGGAGCCCAGGTGGGTGGATCCATCTCCTCTGGCTCCTCCGCCTCCAGTGTC-3'	(G609G)
***DTL*-C37:**	5'-AGCAGCTCGGGAGCCCAGGTGGGTGGATCCATCTCCTCTGGCTCCTCCGCCTCCAGTGTC-3'	(G609G)
	5'-AGCAGCTCGGGAGCCCAGGTGGGTGGATCCATCTCCTCTGGCTCCTCCGCCTCCAGTGTC-3'	(G609G)

b

***R12I* mutant cell colonies**

RAG1	5'-AGGGGAAACCCTCGCTGGAGCAATCTCCAGCAGTCCTGGACAAGCCTGGTGGTCAGAAGTCAGC-3'	**Wild-type**
**R12I-C22**	5'-AGGGGAAACCCTCGCTGGAGTAATCTCCAGCAGTCCTGGACAAGCCTGGTGGTCAGAAGTCAGC-3'	(Q61STOP)
**R12I-C29**	5'-AGGGGAAACCCTCGCTGGAGCAATCTCCAGCAGTCCTGGACAAGCCTGGTGGTCAGAAGTCAGC-3'	Wild-type
**R12I-C56**	5'-AGGGGAAACCCTCGCTGGAGCAATCTCCAGCAGTCCTGGACAAGCCTGGTGGTCAGAAGTCAGC-3'	Wild-type
**R12I-C108**	5'-AGGGGAAACCCTCGCTGGAGCAATCTCCAGCAGTCCTGGACAAGCCTGGTGGTCAGAAGTCAGC-3'	Wild-type
	5'-AGGGGAAACCCTCGCTGGAGTAATCTCCAGCAGTCCTGGACAAGCCTGGTGGTCAGAAGTCAGC-3'	(Q61STOP)
RAG2	5'-CCCACTGTCAGAAAATGTCACTACAGATGATAACAGTTGGTAATAACATGGCCTTAATTCAGCC-3'	**Wild-type**
**R12I-C22**	5'-CCCACTGTCAGAAAATGTCATTAAAGATGATAACAGTTGGTAATAACATGGCCTTAATTCAGCC-3'	(L3L, Q4K)
	5'-CCCACTGTCAGAAAATGTTATTATAGATGATAACAGTTGGTAATAACATGGCCTTAATTCAGCC-3'	(S2L, L3L, Q4STOP)
**R12I-C29**	5'-CCCACTGTCAGAAAATGTTACTACAGATGATAACAGTTGGTAATAACATGGCCTTAATTCAGCC-3'	(S2L)
	5'-CCCACTGTCAGAAAATGTTATTACAGATGATAACAGTTGGTAATAACATGGCCTTAATTCAGCC-3'	(S2L, L3L)
**R12I-C56**	5'-CCCACTGTCAGAAAATGTTATTACAGATGATAACAGTTGGTAATAACATGGCCTTAATTCAGCC-3'	(S2L, L3L)
	5'-CCCACTGTCAGAAAATGTTATTATAGATGATAACAGTTGGTAATAACATGGCCTTAATTCAGCC-3'	(S2L, L3L, Q4STOP)
**R12I-C108**	5'-CCCACTGTCAGAAAATGTTATTATAGATGATAACAGTTGGTAATAACATGGCCTTAATTCAGCC-3'	(S2L, L3L, Q4STOP)
IL2RG	5'-CTACCAAACATTTGTTGTCCAGCTCCAGGACCCACGGGAACCCAGGAGGCAGGACCCACAGACG-3'	**Wild-type**
**R12I-C22**	5'-CTACCAAACATTTGTTGTTTAGCTCCAGGACCCACGGGAACCCAGGAGGCAGGACCCACAGACG-3'	(V154V, Q155STOP)
**R12I-C29**	5'-CTACCAAACATTTGTTGTCCAGCTCCAGGACCCACGGGAACCCAGGAGGCAGGACCCACAGACG-3'	Wild-type
	5'-CTACCAAACATTTGTTGTTTAGCTCCAGGACCCACGGGAACCCAGGAGGCAGGACCCACAGACG-3'	(V154V, Q155STOP)
**R12I-C56**	5'-CTACCAAACATTTGTTGTCCAGCTCCAGGACCCACGGGAACCCAGGAGGCAGGACCCACAGACG-3'	Wild-type
	5'-CTACCAAACATTTGTTGTTTAGCTTTAGGACCCACGGGAACCCAGGAGGCAGGACCCACAGACG-3'	(V154V, Q155STOP, L156L, Q157STOP)
**R12I-C108**	5'-CTACCAAACATTTGTTGTTTAGCTCCAGGACCCACGGGAACCCAGGAGGCAGGACCCACAGACG-3'	(V154V, Q155STOP)

Supplementary Fig. 4 Simultaneous base editing of *DMD*, *TYR*, and *LMNA* or *RAG1*, *RAG2*, and *IL2RG* genes in porcine fibroblast cells. a and **b** T-cloning and Sanger sequencing of base editing of *DMD*, *TYR*, and *LMNA* in individual cell colony 1#, 23#, 37# (**a**), and *RAG1*, *RAG2*, and *IL2RG* in cell colony 22#, 29#, 56#, 108# (**b**).

Supplementary Fig. 5

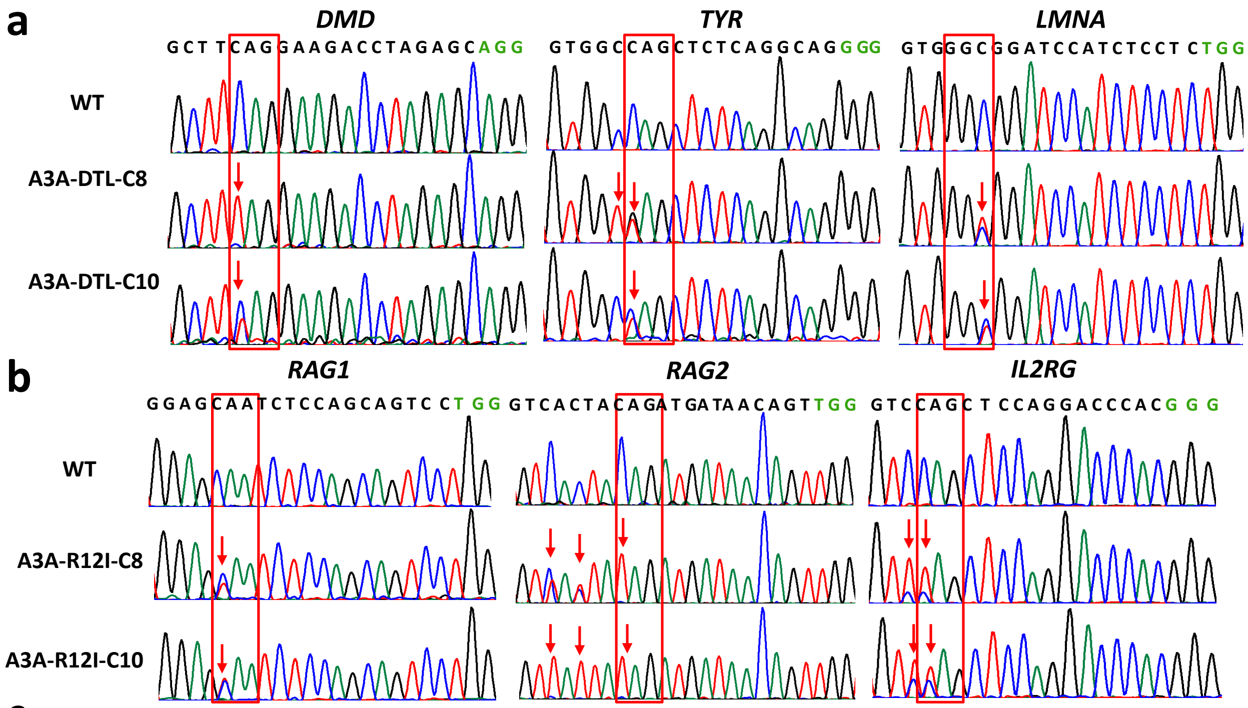

c

DMD 5'-TTTTTTTATCTTTCAGGTGCTTCAGGAAGACCTAGAGCAGGAACAAGTCAGGGTCAATTC-3' **Wild-type**
DTL-C8: 5'-TTTTTTTATCTTTCAGGTGCTTTAGGAAGACCTAGAGCAGGAACAAGTCAGGGTCAATTC-3' (Q493STOP)
DTL-C10: 5'-TTTTTTTATCTTTCAGGTGCTTCAGGAAGACCTAGAGCAGGAACAAGTCAGGGTCAATTC-3' Wild-type
5'-TTTTTTTATCTTTCAGGTGCTTTAGGAAGACCTAGAGCAGGAACAAGTCAGGGTCAATTC-3' (Q493STOP)

TYR 5'-GAGGTGATGGGAGTCCCTGTGGCCAGCTCTCAGGCAGGGGTTCCTGTCAGGACATCATTC-3' **Wild-type**
DTL-C8: 5'-GAGGTGATGGGAGTCCCTGTGGTTAGCTCTCAGGCAGGGGTTCCTGTCAGGACATCATTC-3' (13/20) (G27G, Q28STOP)
5'-GAGGTGATGGGAGTCCCTGTGGTGAGCTCTTAGGCAGGGGTTCCTGTCAGGACATCATTC-3' (7/20) (G27G, S28E)
DTL-C10: 5'-GAGGTGATGGGAGTCCCTGTGGCCAGCTCTCAGGCAGGGGTTCCTGTCAGGACATCATTC-3' **Wild-type**
5'-GAGGTGATGGGAGTCCCTGTGGCTAGCTCTCAGGCAGGGGTTCCTGTCAGGACATCATTC-3' (Q28STOP)

LMNA 5'-AGCAGCTCGGGAGCCCAGGTGGGCGGATCCATCTCCTCTGGCTCCTCCGCCTCCAGTGTC-3' **Wild-type**
DTL-C8: 5'-AGCAGCTCGGGAGCCCAGGTGGGCGGATCCATCTCCTCTGGCTCCTCCGCCTCCAGTGTC-3' Wild-type
5'-AGCAGCTCGGGAGCCCAGGTGGGTGGATCCATCTCCTCTGGCTCCTCCGCCTCCAGTGTC-3' (G608G)
DTL-C10: 5'-AGCAGCTCGGGAGCCCAGGTGGGCGGATCCATCTCCTCTGGCTCCTCCGCCTCCAGTGTC-3' Wild-type
5'-AGCAGCTCGGGAGCCCAGGTGGGTGGATCCATCTCCTCTGGCTCCTCCGCCTCCAGTGTC-3' (G608G)

d

RAG1 5'-AGGGGAAACCCTCGCTGGAGCAATCTCCAGCAGTCCTGGACAAGCCTGGTGGTCAGAAG-3' **Wild-type**
R12I-C8: 5'-AGGGGAAACCCTCGCTGGAGCAATCTCCAGCAGTCCTGGACAAGCCTGGTGGTCAGAAG-3' Wild-type
5'-AGGGGAAACCCTCGCTGGAGTAATCTCCAGCAGTCCTGGACAAGCCTGGTGGTCAGAAG-3' (Q61STOP)
R12I-C10: 5'-AGGGGAAACCCTCGCTGGAGCAATCTCCAGCAGTCCTGGACAAGCCTGGTGGTCAGAAG-3' Wild-type
5'-AGGGGAAACCCTCGCTGGAGTAATTTCCAGCAGTCCTGGACAAGCCTGGTGGTCAGAAG-3' (Q61STOP)

RAG2 5'-CCCACTGTCAGAAAATGTCACTACAGATGATAACAGTTGGTAATAACATGGCCTTAATT-3' **Wild-type**
R12I-C8: 5'-CCCACTGTCAGAAAATGTTATTATAGATGATAACAGTTGGTAATAACATGGCCTTAATT-3' (S2L,L3L,Q4STOP)
5'-CCCACTGTCAGAAAATGTCACTATAGATGATAACAGTTGGTAATAACATGGCCTTAATT-3' (Q4STOP)
R12I-C10: 5'-CCCACTGTCAGAAAATGTTATTATAGATGATAACAGTTGGTAATAACATGGCCTTAATT-3' S2L,L3L,Q4STOP)

IL2RG 5'-CTACCAAACATTTGTTGTCCAGCTCCAGGACCCACGGGAACCCAGGAGGCAGGACCCAC-3' **Wild-type**
R12I-C8: 5'-CTACCAAACATTTGTTGTCCAGCTCCAGGACCCACGGGAACCCAGGAGGCAGGACCCAC-3' Wild-type
5'-CTACCAAACATTTGTTGTTTAGCTCCAGGACCCACGGGAACCCAGGAGGCAGGACCCAC-3' (V154V,Q155STOP)
R12I-C10: 5'-CTACCAAACATTTGTTGTCCAGCTCCAGGACCCACGGGAACCCAGGAGGCAGGACCCAC-3' Wild-type
5'-CTACCAAACATTTGTTGTTTAGCTCCAGGACCCACGGGAACCCAGGAGGCAGGACCCAC-3' (V154V,Q155STOP)

e

No. of screened colonies	No. of colonies with single gene base editing (%)			No. of colonies with two genes base editing (%)			No. of colonies with three genes base editing (%)
	DMD	TYR	LMNA	DMD and TYR	TYR and LMNA	DMD and LMNA	DMD, TYR and LMNA
20	0	0	0	1 (5.0)	1 (5.0)	3 (15.0)	11 (55.0)
	RAG1	*RAG2*	*IL2RG*	*RAG1* and *RAG2*	*RAG1* and *IL2RG*	*RAG2* and *IL2RG*	*RAG1*, *RAG2* and *IL2RG*
20	2 (10.0)	2 (10.0)	1 (5.0)	0	0	2 (10.0)	8 (40.0)

Supplementary Fig. 5 hA3A-BE3-mediated multiple genes base editing in porcine somatic cells. a and **b** Sanger sequencing results of the target sites in cell colony A3A-DTL-C8, C10 (**a**) and A3A-R12I-C8, C10 (**b**). **c** and **d** T-cloning and Sanger sequencing of base editing of A3A-DTL-C8, C10 (**c**) and A3A-R12I-C8, C10 (**d**). **e** Summary of multiple sites base editing by hA3A-BE3 in porcine somatic cells

Supplementary Fig. 6

a

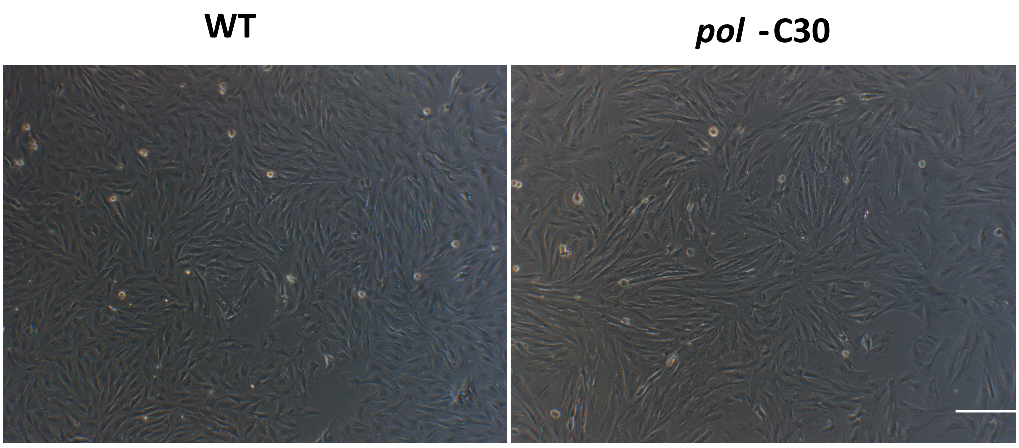

b

		Val (V)			Gln(Q)			Arg(R)			Leu(L)			Ile (I)			Gln(Q)			Gln(Q)				
		G	T	T	C	A	A	A	G	A	T	T	A	A	T	C	C	A	A	C	A	G	G	G
pol-C30	T	0.0	100.0	100.0	84.9	0.0	0.0	0.0	0.0	0.0	100.0	100.0	0.0	0.0	100.0	2.0	0.0	0.0	0.0	0.0	0.0	0.0	0.0	0.0
	C	0.0	0.0	0.0	13.2	0.0	0.0	0.0	0.0	0.0	0.0	0.0	0.0	0.0	0.0	98.0	100.0	0.0	0.0	100.0	0.0	0.0	0.0	0.0
	G	100.0	0.0	0.0	1.9	0.0	0.0	0.0	100.0	0.0	0.0	0.0	0.0	0.0	0.0	0.0	0.0	0.0	0.0	0.0	0.0	100.0	100.0	100.0
	A	0.0	0.0	0.0	0.0	100.0	100.0	100.0	0.0	100.0	0.0	0.0	100.0	100.0	0.0	0.0	0.0	100.0	100.0	0.0	100.0	0.0	0.0	0.0
pol-C87	T	0.0	100.0	100.0	84.0	0.0	0.0	0.0	0.0	0.0	100.0	100.0	0.0	0.0	100.0	2.4	0.0	0.0	0.0	0.0	0.0	0.0	0.0	0.0
	C	0.0	0.0	0.0	13.6	0.0	0.0	0.0	0.0	0.0	0.0	0.0	0.0	0.0	0.0	97.6	100.0	0.0	0.0	100.0	0.0	0.0	0.0	0.0
	G	100.0	0.0	0.0	2.4	0.0	0.0	0.0	100.0	0.0	0.0	0.0	0.0	0.0	0.0	0.0	0.0	0.0	0.0	0.0	0.0	100.0	100.0	100.0
	A	0.0	0.0	0.0	0.0	100.0	100.0	100.0	0.0	100.0	0.0	0.0	100.0	100.0	0.0	0.0	0.0	100.0	100.0	0.0	100.0	0.0	0.0	0.0

c

Gene	No. of screened colonies	No. of colonies with base editing (<50%)	No. of colonies with base editing (≥50%)
pol	155	40 (25.8%)	59 (38.1%)

Supplementary Fig. 4 Base editing of the *pol* gene in porcine somatic cells. a Photograph of selected *pol*-inactivated and WT colony. Scale bars: 20 µm. **b** Summary of the deep sequencing of the on-target sites of the *pol* gene in cell colony 30# and 87#. **c** Summary of base editing efficiency of selected cell colonies.

Supplementary Fig. 7

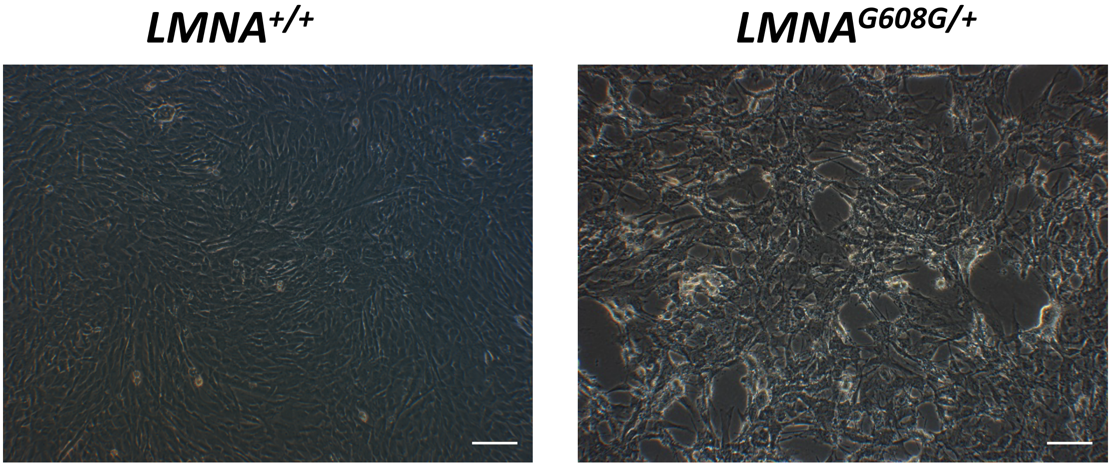

Supplementary Fig. 7 Photographs of the *LMNA*$^{+/+}$ and *LMNA*$^{G608G/+}$ cell colonies. *LMNA*$^{G608G/+}$ cell colony exhibited premature senescence phenotypes. Scale bars: 20 μm.

Supplementary Fig. 8

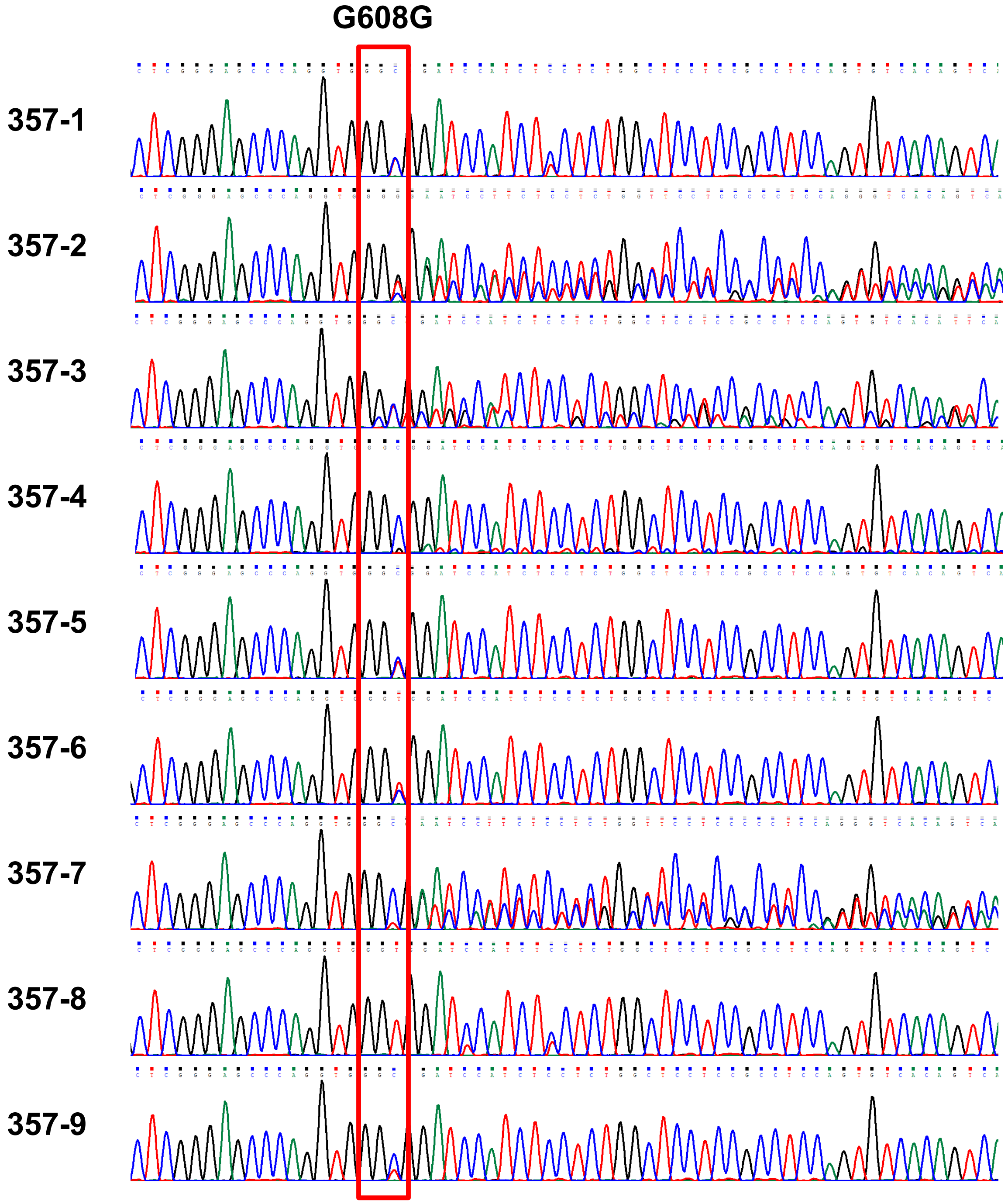

Supplementary Fig. 8 Sanger sequencing results of *LMNA*^{G608G} mutant piglets

Supplementary Fig. 9

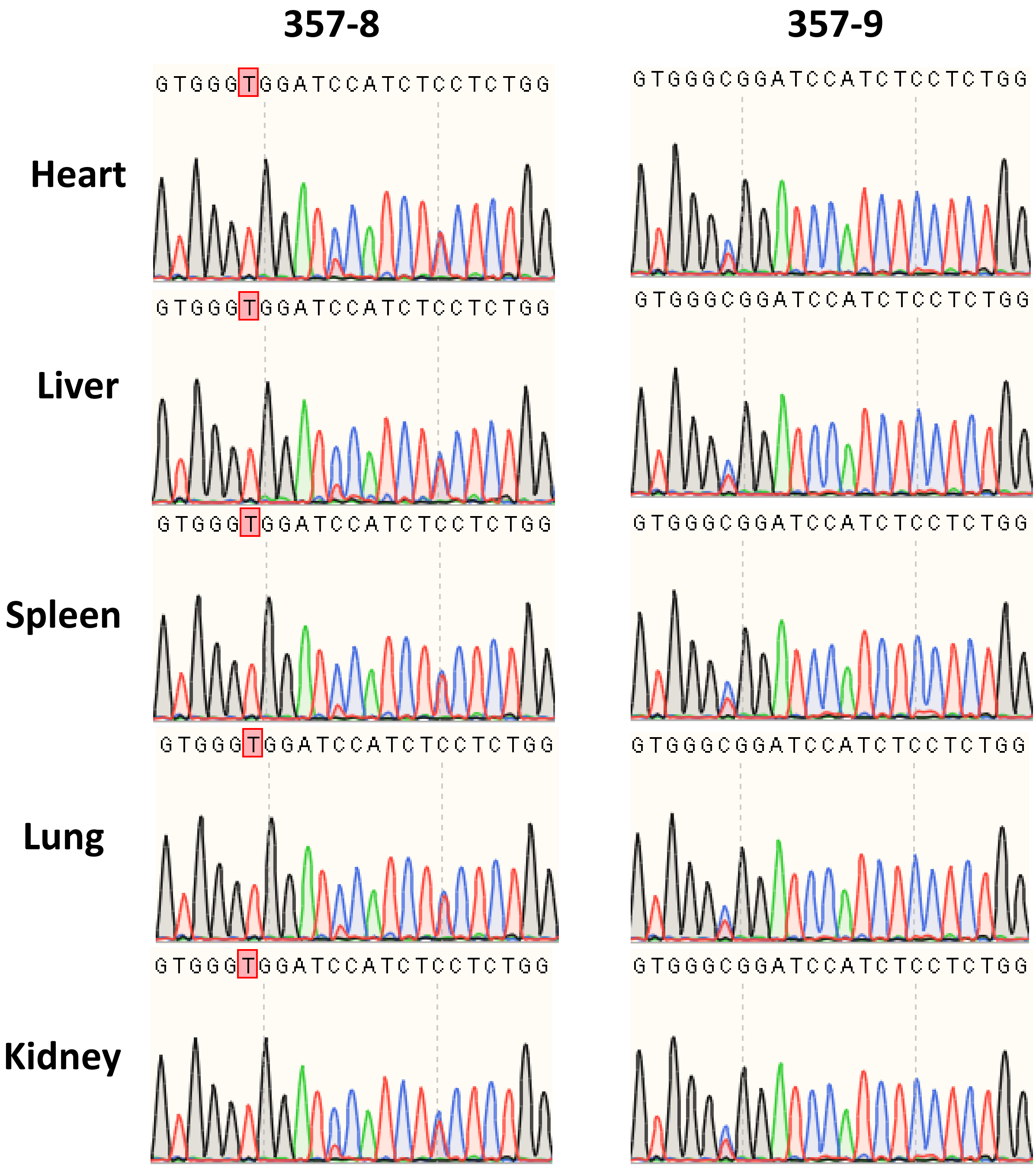

Supplementary Fig. 9 *LMNA* c.1824C > T mutations in the heart, liver, spleen, lung and kidney of 357-8 and 357-9 piglets were detected by Sanger sequencing

Supplementary Fig. 10

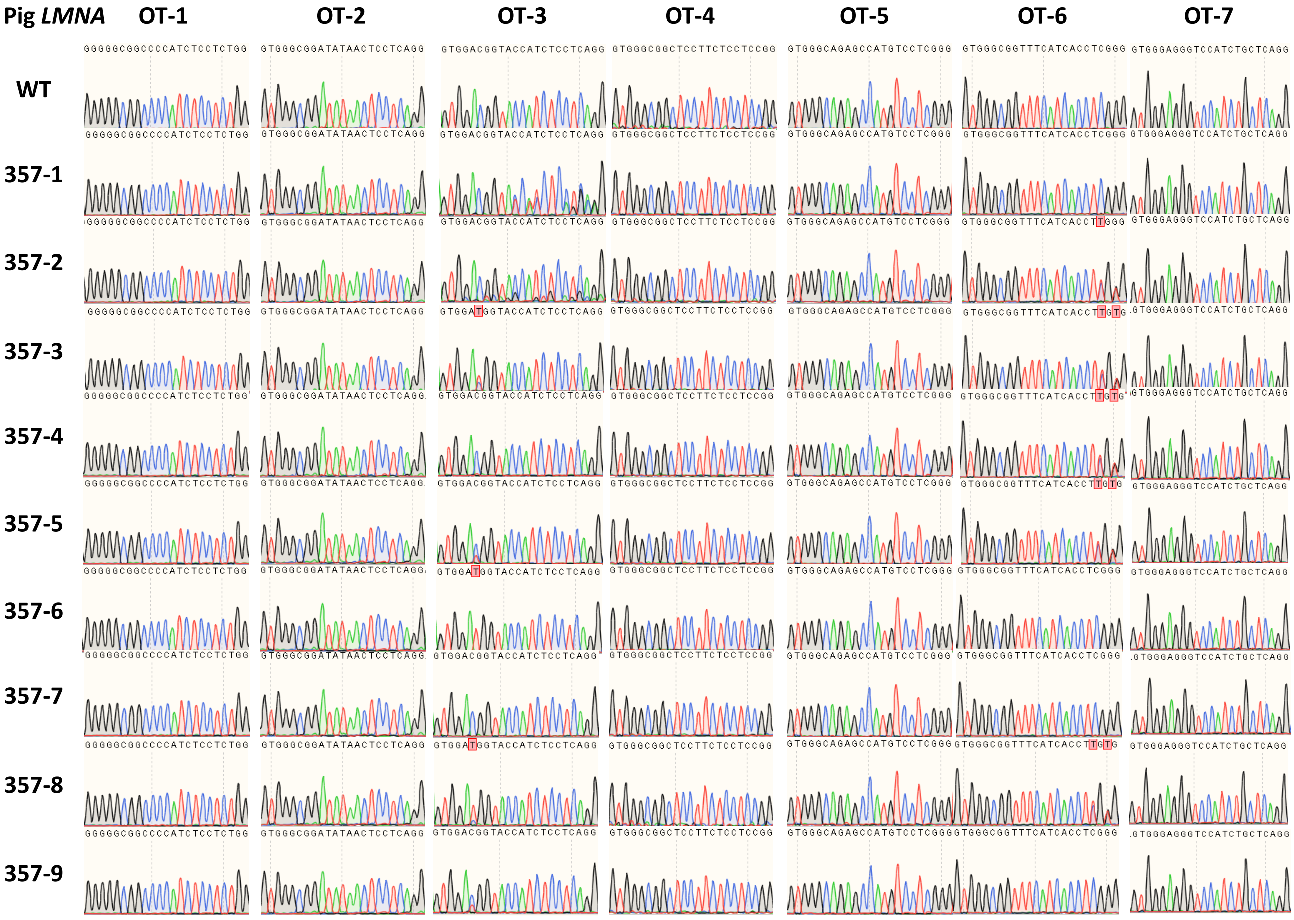

Supplementary Fig. 15 Off-target analysis of *LMNA*^{G608G} mutant piglets.

Supplementary Fig. 11

***DMD* mutant colonies**

	TTTTTATCTTTCAGGT**GCTTCAGGAAGACCTAGAGCAGG**AACAAGTCAGGGTCAATTCTCTCAC	**Wild-type**
***DMD*-C3:**	TTTTTATCTTTCAGGT------------------GCAGGAACAAGTCAGGGTCAATTCTCTCAC	(Δ18)
	TTTTTATCTTTCAGGTGCTT**T**AGGAAGACCTAGAGCAGGAACAAGTCAGGGTCAATTCTCTCAC	(Q493STOP)
***DMD*-C17:**	TTTTTATCTTTCAGGTGCTTCAGGAAGACCTAGAGCAGGAACAAGTCAGGGTCAATTCTCTCAC	Wild-type
	TTTTTATCTTTCAGGTGCTT**T**AGGAAGACCTAGAGCAGGAACAAGTCAGGGTCAATTCTCTCAC	(Q493STOP)
***DMD*-C36:**	TTTTTATCTTTCAGGT------------------GCAGGAACAAGTCAGGGTCAATTCTCTCAC	(Δ18)
	TTTTTATCTTTCAGGTGCTT**T**AGGAAGACCTAGAGCAGGAACAAGTCAGGGTCAATTCTCTCAC	(Q493STOP)
***DMD*-C59:**	TTTTTATCTTTCAGGT------------------GCAGGAACAAGTCAGGGTCAATTCTCTCAC	(Δ18)
	TTTTTATCTTTCAGGTGCTT**T**AGGAAGACCTAGAGCAGGAACAAGTCAGGGTCAATTCTCTCAC	(Q493STOP)
***DMD*-C67:**	TTTTTATCTTTCAGGTGCTTCAGGAAGACCTAGAGCAGGAACAAGTCAGGGTCAATTCTCTCAC	Wild-type
	TTTTTATCTTTCAGGTGCTT**T**AGGAAGACCTAGAGCAGGAACAAGTCAGGGTCAATTCTCTCAC	(Q493STOP)
***DMD*-C87:**	TTTTTATCTTTCAGGT------------------GCAGGAACAAGTCAGGGTCAATTCTCTCAC	(Δ18)
	TTTTTATCTTTCAGGTGCTT**T**AGGAAGACCTAGAGCAGGAACAAGTCAGGGTCAATTCTCTCAC	(Q493STOP)
***DMD*-C92:**	TTTTTATCTTTCAGGTGCTTCAGGAAGACCTAGAGCAGGAACAAGTCAGGGTCAATTCTCTCAC	Wild-type
	TTTTTATCTTTCAGGTGCTT**T**AGGAAGACCTAGAGCAGGAACAAGTCAGGGTCAATTCTCTCAC	(Q493STOP)

Supplementary Fig. 11 Genotype of selected cell colonies for SCNT to generate *DMD* heterozygous piglets.

Supplementary Fig. 12

a

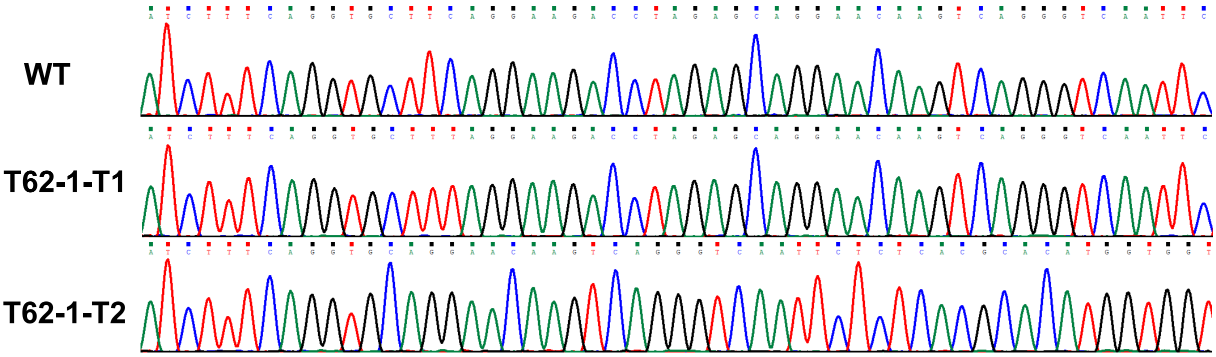

b

***DMD* mutant piglets**

```
           TTTTTATCTTTCAGGTGCTTCAGGAAGACCTAGAGCAGGAACAAGTCAGGGTCAATTCTCTCAC Wild-type
T62-1-1:   TTTTTATCTTTCAGGT------------------GCAGGAACAAGTCAGGGTCAATTCTCTCAC (Δ18)
           TTTTTATCTTTCAGGTGCTTTAGGAAGACCTAGAGCAGGAACAAGTCAGGGTCAATTCTCTCAC (Q493STOP)
```

Supplementary Fig. 12 Genotype of newborn *DMD* mutant piglet. a and **b** T-cloning and Sanger sequencing of base editing of *DMD* in newborn T62-1-1 piglet.

Supplementary Fig. 13

R121 mutant piglets

RAG1	5′-AGGGGAAACCCTCGCTGGAGCAATCTCCAGCAGTCCTGGACAAGCCTGGTGGTCAGAAGTCAGC-3′	Wild-type
A632-1	5′-AGGGGAAACCCTCGCTGGAGTAATCTCCAGCAGTCCTGGACAAGCCTGGTGGTCAGAAGTCAGC-3′	Q61STOP
A632-2	5′-AGGGGAAACCCTCGCTGGAGCAATCTCCAGCAGTCCTGGACAAGCCTGGTGGTCAGAAGTCAGC-3′	Wild-type
	5′-AGGGGAAACCCTCGCTGGAGTAATCTCCAGCAGTCCTGGACAAGCCTGGTGGTCAGAAGTCAGC-3′	(Q61STOP)
A632-3	5′-AGGGGAAACCCTCGCTGGAGCAATCTCCAGCAGTCCTGGACAAGCCTGGTGGTCAGAAGTCAGC-3′	Wild-type
A633-1	5′-AGGGGAAACCCTCGCTGGAGCAATCTCCAGCAGTCCTGGACAAGCCTGGTGGTCAGAAGTCAGC-3′	Wild-type
A633-2	5′-AGGGGAAACCCTCGCTGGAGCAATCTCCAGCAGTCCTGGACAAGCCTGGTGGTCAGAAGTCAGC-3′	Wild-type
RAG2	5′-CCCACTGTCAGAAAATGTCACTACAGATGATAACAGTTGGTAATAACATGGCCTTAATTCAGCC-3′	Wild-type
A632-1	5′-CCCACTGTCAGAAAATGTCATTAAAGATGATAACAGTTGGTAATAACATGGCCTTAATTCAGCC-3′	L3L, Q4K
	5′-CCCACTGTCAGAAAATGTTATTATAGATGATAACAGTTGGTAATAACATGGCCTTAATTCAGCC-3′	S2L, L3L, Q4STOP
A632-2	5′-CCCACTGTCAGAAAATGTTATTATAGATGATAACAGTTGGTAATAACATGGCCTTAATTCAGCC-3′	S2L, L3L, Q4STOP
A632-3	5′-CCCACTGTCAGAAAATGTTACTACAGATGATAACAGTTGGTAATAACATGGCCTTAATTCAGCC-3′	S2L
	5′-CCCACTGTCAGAAAATGTTATTACAGATGATAACAGTTGGTAATAACATGGCCTTAATTCAGCC-3′	S2L, L3L
A633-1	5′-CCCACTGTCAGAAAATGTTATTACAGATGATAACAGTTGGTAATAACATGGCCTTAATTCAGCC-3′	S2L, L3L
	5′-CCCACTGTCAGAAAATGTTATTATAGATGATAACAGTTGGTAATAACATGGCCTTAATTCAGCC-3′	S2L, L3L, Q4STOP
A633-2	5′-CCCACTGTCAGAAAATGTTATTACAGATGATAACAGTTGGTAATAACATGGCCTTAATTCAGCC-3′	S2L, L3L
	5′-CCCACTGTCAGAAAATGTTATTATAGATGATAACAGTTGGTAATAACATGGCCTTAATTCAGCC-3′	S2L, L3L, Q4STOP
IL2RG	5′-CTACCAAACATTTGTTGTCCAGCTCCAGGACCCACGGGAACCCAGGAGGCAGGACCCACAGACG-3′	Wild-type
A632-1	5′-CTACCAAACATTTGTTGTTTAGCTCCAGGACCCACGGGAACCCAGGAGGCAGGACCCACAGACG-3′	V154V, Q155STOP
A632-2	5′-CTACCAAACATTTGTTGTTTAGCTCCAGGACCCACGGGAACCCAGGAGGCAGGACCCACAGACG-3′	V154V, Q155STOP
A632-3	5′-CTACCAAACATTTGTTGTCCAGCTCCAGGACCCACGGGAACCCAGGAGGCAGGACCCACAGACG-3′	Wild-type
	5′-CTACCAAACATTTGTTGTTTAGCTCCAGGACCCACGGGAACCCAGGAGGCAGGACCCACAGACG-3′	V154V, Q155STOP
A633-1	5′-CTACCAAACATTTGTTGTCCAGCTCCAGGACCCACGGGAACCCAGGAGGCAGGACCCACAGACG-3′	Wild-type
	5′-CTACCAAACATTTGTTGTTTAGCTTTAGGACCCACGGGAACCCAGGAGGCAGGACCCACAGACG-3′	V154V, Q155STOP, L156L, Q157STOP
A633-2	5′-CTACCAAACATTTGTTGTCCAGCTCCAGGACCCACGGGAACCCAGGAGGCAGGACCCACAGACG-3′	Wild-type
	5′-CTACCAAACATTTGTTGTTTAGCTTTAGGACCCACGGGAACCCAGGAGGCAGGACCCACAGACG-3′	V154V, Q155STOP, L156L, Q157STOP

Supplementary Fig. 13 Genotype of newborn *RAG1*, *RAG2*, and *IL2RG* mutant piglets.

Supplementary Fig. 14

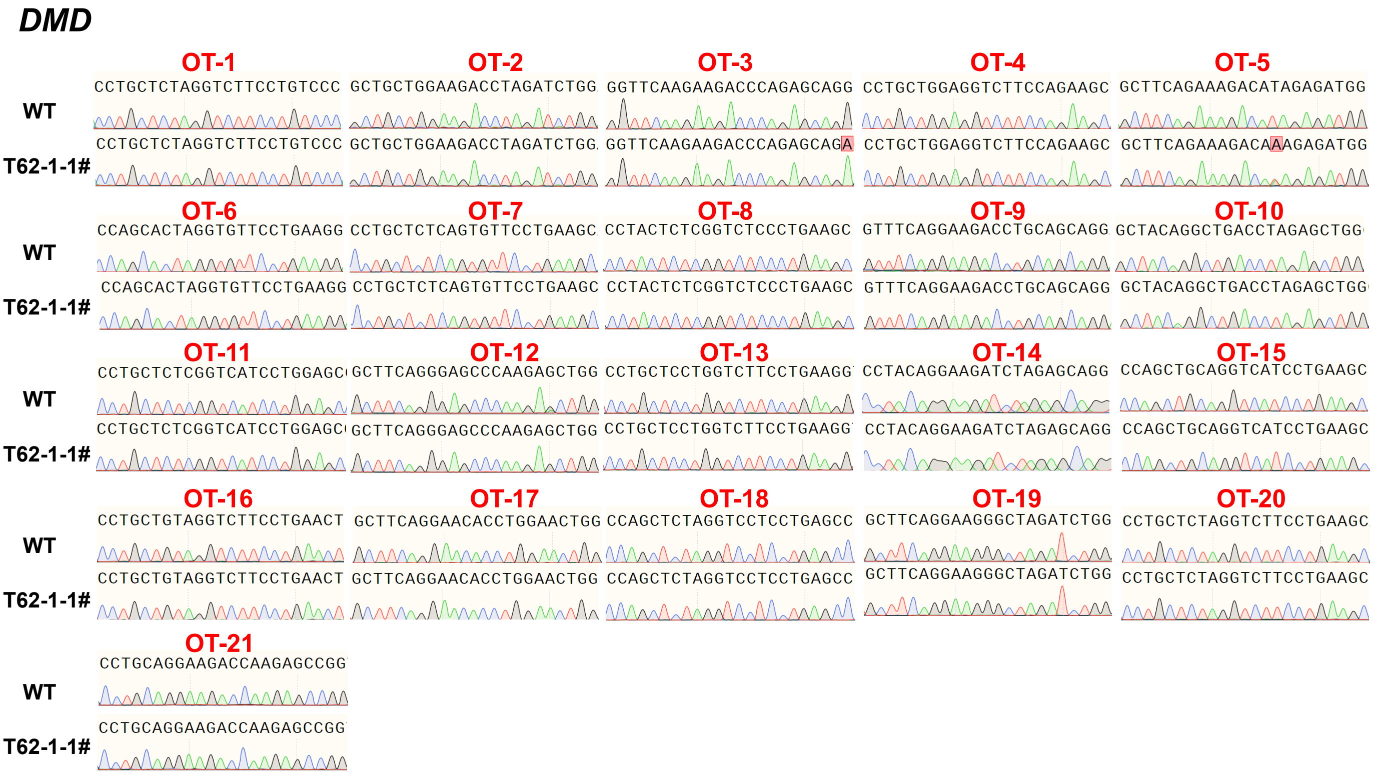

Supplementary Fig. 14 Off-target analysis of *DMD* mutant piglet (T62-1-1).

Supplementary Fig. 15

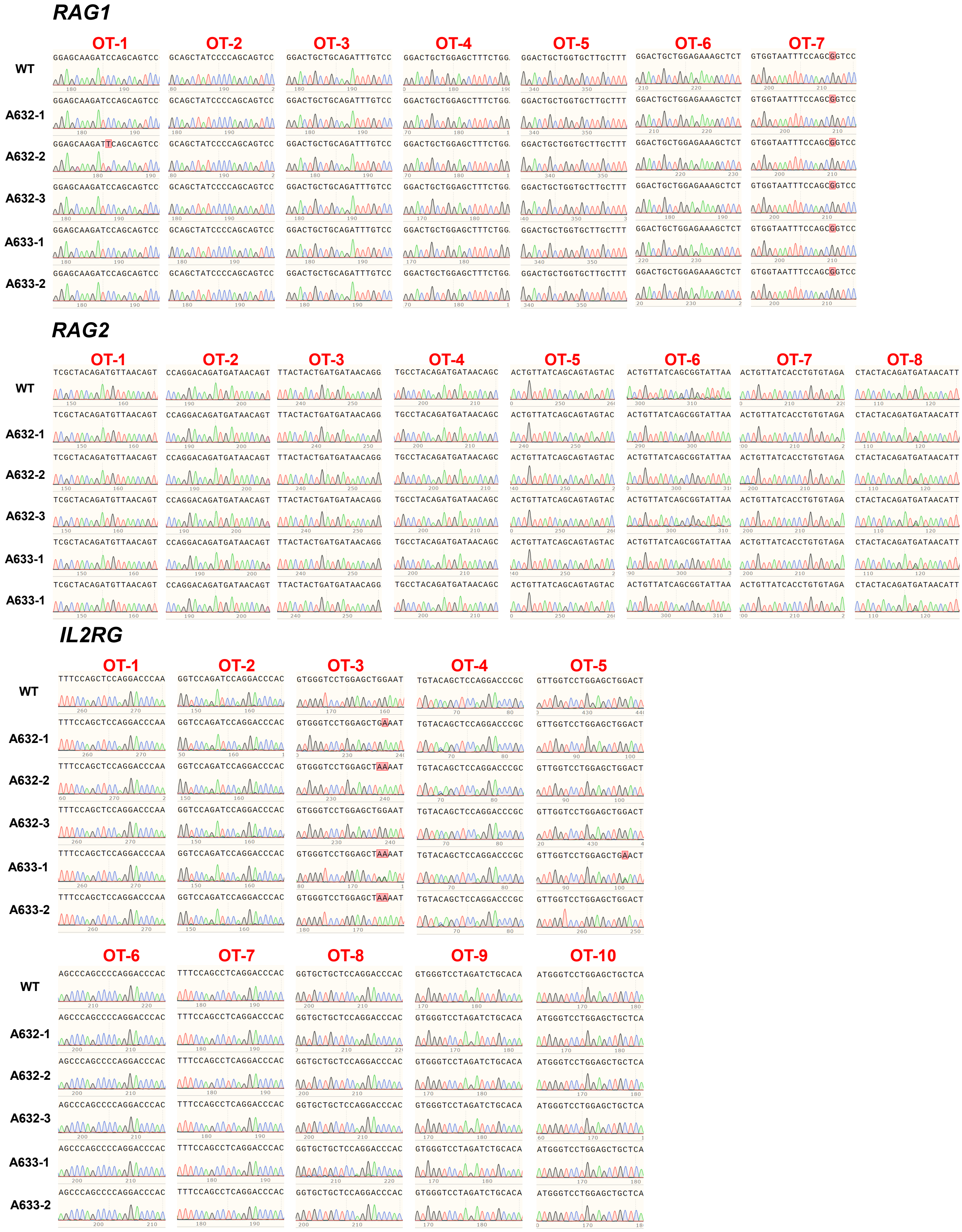

Supplementary Fig. 15 Off-target analysis of *RAG1*, *RAG2*, and *IL2RG* mutant piglets.

Supplementary Fig. 16

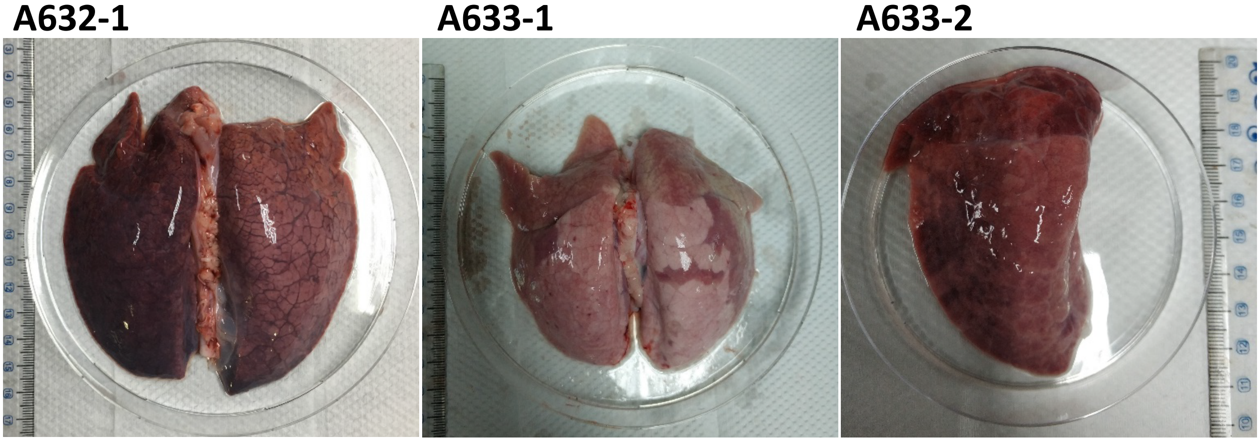

Supplementary Fig. 16 Photographs of the lungs from piglets A632-1, A633-1, and A633-2, died 49, 41, and 74 days after birth.

Supplementary Fig. 17

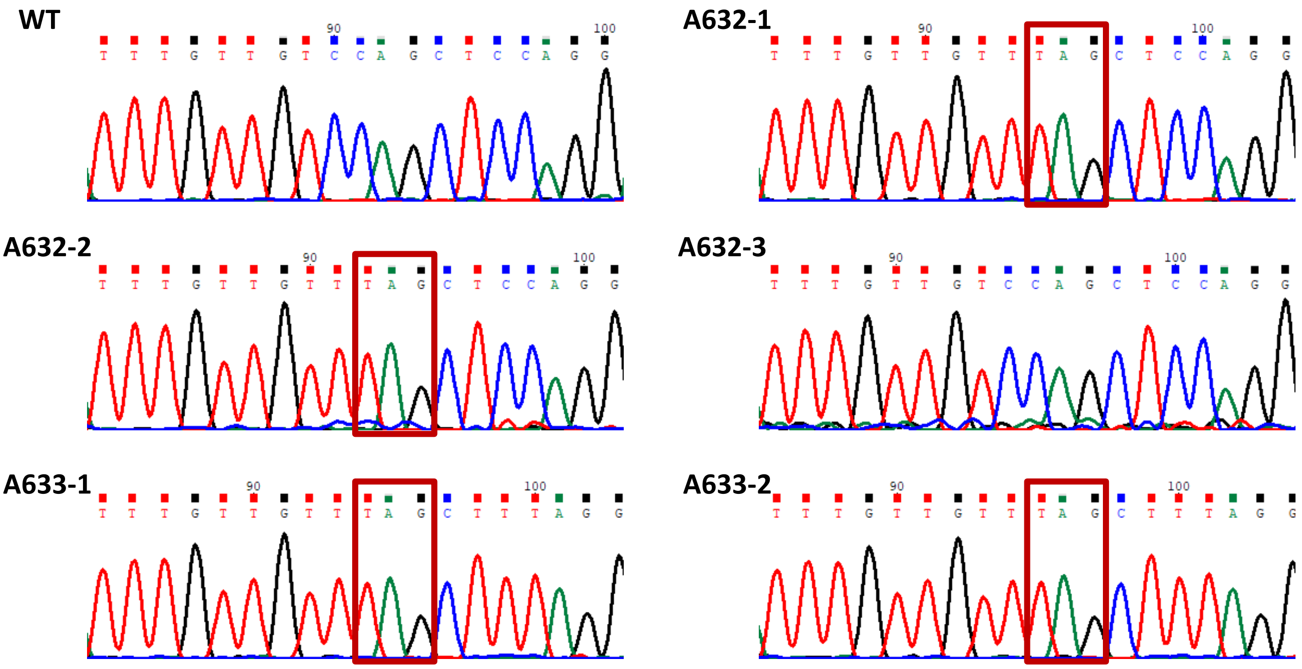

Supplementary Fig. 17 RT-PCR and Sanger sequencing of *IL2RG* mRNA in peripheral blood of A632-1, A632-2, A632-3, A633-1, A633-2, and WT piglets.
