# Supplementary material for: Efficient base editing for multiple genes and loci in pigs using base editors
Source: Nat Commun. 2019 Jun 28;10:2852. doi: 10.1038/s41467-019-10421-8 (PMC6599043; doi:10.1038/s41467-019-10421-8)
Supplement: Supplementary file 3 — Description of Additional Supplementary Files [file 41467_2019_10421_MOESM3_ESM.pdf]

nature research

Corresponding author(s): Liangxue Lai and Kepin Wang

Last updated by author(s): May 6, 2019

# Reporting Summary

Nature Research wishes to improve the reproducibility of the work that we publish. This form provides structure for consistency and transparency in reporting. For further information on Nature Research policies, see Authors & Referees and the Editorial Policy Checklist.

## Statistics

For all statistical analyses, confirm that the following items are present in the figure legend, table legend, main text, or Methods section.

| n/a | Confirmed | |
|---|---|---|
| ☐ | ☒ | The exact sample size (*n*) for each experimental group/condition, given as a discrete number and unit of measurement |
| ☐ | ☒ | A statement on whether measurements were taken from distinct samples or whether the same sample was measured repeatedly |
| ☒ | ☐ | The statistical test(s) used AND whether they are one- or two-sided<br>*Only common tests should be described solely by name; describe more complex techniques in the Methods section.* |
| ☒ | ☐ | A description of all covariates tested |
| ☐ | ☒ | A description of any assumptions or corrections, such as tests of normality and adjustment for multiple comparisons |
| ☐ | ☒ | A full description of the statistical parameters including central tendency (e.g. means) or other basic estimates (e.g. regression coefficient) AND variation (e.g. standard deviation) or associated estimates of uncertainty (e.g. confidence intervals) |
| ☒ | ☐ | For null hypothesis testing, the test statistic (e.g. *F*, *t*, *r*) with confidence intervals, effect sizes, degrees of freedom and *P* value noted<br>*Give P values as exact values whenever suitable.* |
| ☒ | ☐ | For Bayesian analysis, information on the choice of priors and Markov chain Monte Carlo settings |
| ☒ | ☐ | For hierarchical and complex designs, identification of the appropriate level for tests and full reporting of outcomes |
| ☐ | ☒ | Estimates of effect sizes (e.g. Cohen's *d*, Pearson's *r*), indicating how they were calculated |

*Our web collection on statistics for biologists contains articles on many of the points above.*

## Software and code

Policy information about availability of computer code

| | |
|---|---|
| Data collection | No software was used. |
| Data analysis | FastQC (v0.11.4), Microsoft Excel (v14.7.7) |

For manuscripts utilizing custom algorithms or software that are central to the research but not yet described in published literature, software must be made available to editors/reviewers. We strongly encourage code deposition in a community repository (e.g. GitHub). See the Nature Research guidelines for submitting code & software for further information.

## Data

Policy information about availability of data

All manuscripts must include a data availability statement. This statement should provide the following information, where applicable:

- Accession codes, unique identifiers, or web links for publicly available datasets
- A list of figures that have associated raw data
- A description of any restrictions on data availability

All date was available in figures, supplementary figures and supplementary tables.

# Field-specific reporting

Please select the one below that is the best fit for your research. If you are not sure, read the appropriate sections before making your selection.

☒ Life sciences ☐ Behavioural & social sciences ☐ Ecological, evolutionary & environmental sciences

For a reference copy of the document with all sections, see nature.com/documents/nr-reporting-summary-flat.pdf

# Life sciences study design

All studies must disclose on these points even when the disclosure is negative.

| | |
|---|---|
| Sample size | No statistical methods were used to predetermine sample size. In this study, nine target sites were tested in the rabbit or porcine genome, which was sufficient in the field. |
| Data exclusions | No data were excluded. |
| Replication | The experimental findings in all figures were reproduced successfully. |
| Randomization | Samples were not randomized. |
| Blinding | The investigators were not blinded to group allocation. |

# Reporting for specific materials, systems and methods

We require information from authors about some types of materials, experimental systems and methods used in many studies. Here, indicate whether each material, system or method listed is relevant to your study. If you are not sure if a list item applies to your research, read the appropriate section before selecting a response.

## Materials & experimental systems

| n/a | Involved in the study | |
|---|---|---|
| ☐ | ☒ | Antibodies |
| ☐ | ☒ | Eukaryotic cell lines |
| ☒ | ☐ | Palaeontology |
| ☐ | ☒ | Animals and other organisms |
| ☒ | ☐ | Human research participants |
| ☒ | ☐ | Clinical data |

## Methods

| n/a | Involved in the study | |
|---|---|---|
| ☒ | ☐ | ChIP-seq |
| ☐ | ☒ | Flow cytometry |
| ☒ | ☐ | MRI-based neuroimaging |

## Antibodies

| | |
|---|---|
| Antibodies used | Mouse anti-pig CD3e (BD Pharmingen), CD8a (BD Pharmingen), goat anti-pig IgM (AbD Serotec) and Mouse anti-human Lamin A/C Antibody (ab108922, abcam) |
| Validation | All antibody were validated. |

## Eukaryotic cell lines

Policy information about cell lines

| | |
|---|---|
| Cell line source(s) | Porcine fetal fibroblasts were isolated from our lab. |
| Authentication | No cell lines have were authenticated. |
| Mycoplasma contamination | All cell lines have been tested for mycoplasma contamination free by PCR methods. |
| Commonly misidentified lines (See ICLAC register) | No commonly misidentified cell lines were used |

## Animals and other organisms

Policy information about studies involving animals; ARRIVE guidelines recommended for reporting animal research

| | |
|---|---|
| Laboratory animals | Bama miniature pigs (1-30 weeks old, male and female) and large white pigs (1-30 weeks old, male and female) were used in this study. |
| Wild animals | The study did not involve wild animals |
| Field-collected samples | The study did not involve field collected samples. |
| Ethics oversight | All animal studies were approved by the Institutional Animal Care and Use Committees at Guangzhou Institute of Biomedicine and Health Chinese Academy of Sciences (Animal Welfare Assurance #A5748-01) |

Note that full information on the approval of the study protocol must also be provided in the manuscript.

## Flow Cytometry

### Plots

Confirm that:

☒ The axis labels state the marker and fluorochrome used (e.g. CD4-FITC).

☒ The axis scales are clearly visible. Include numbers along axes only for bottom left plot of group (a 'group' is an analysis of identical markers).

☒ All plots are contour plots with outliers or pseudocolor plots.

☒ A numerical value for number of cells or percentage (with statistics) is provided.

### Methodology

| | |
|---|---|
| Sample preparation | Anticoagulated peripheral blood (PB) was obtained from RAG1, RAG2, and IL2RG gene-modified pigs and age-matched control pigs. Red blood cells were eliminated with lysis buffer. |
| Instrument | Accuri C6 flow cytometer (Accuri Cytometers, Ann Arbor, MI). |
| Software | Data acquisition: BD FACSdiva 8.0.1. Data analysis: Flow Jo X. |
| Cell population abundance | Red blood cells were eliminated with lysis buffer and mononuclear cells were analyzed. |
| Gating strategy | We selected the mononuclear cells as a gate for the analysis. |

☒ Tick this box to confirm that a figure exemplifying the gating strategy is provided in the Supplementary Information.
