# Supplementary material for: Efficient base editing for multiple genes and loci in pigs using base editors
Source: Nat Commun. 2019 Jun 28;10:2852. doi: 10.1038/s41467-019-10421-8 (PMC6599043; doi:10.1038/s41467-019-10421-8)
Supplement: Supplementary file 8 — Source Data [file 41467_2019_10421_MOESM8_ESM.zip › Source Data of NCOMMS-18-32570A/Uncropped versions of gels or blots.pptx]

## Slide 1
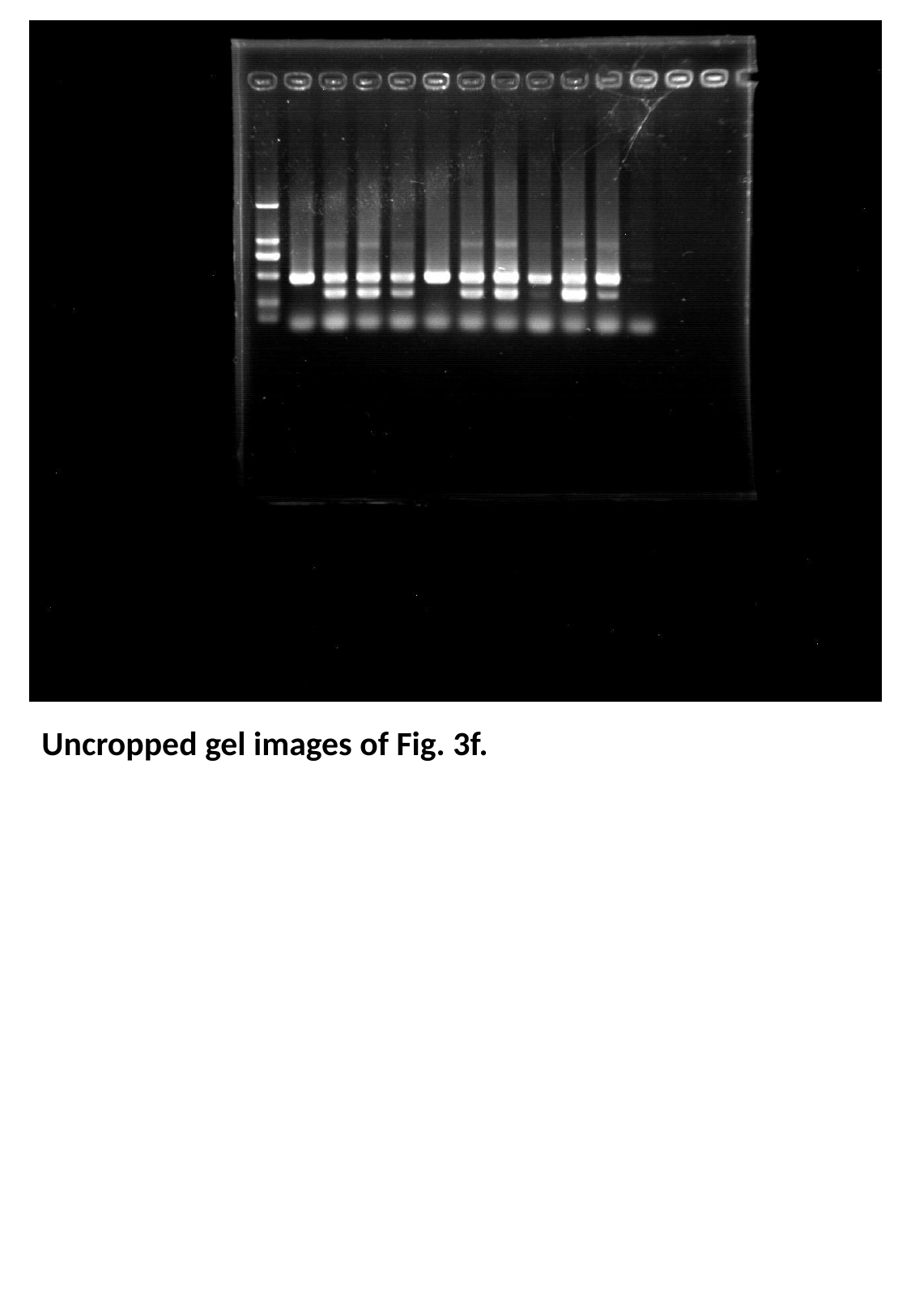

Uncropped gel images of Fig. 3f.

## Slide 2
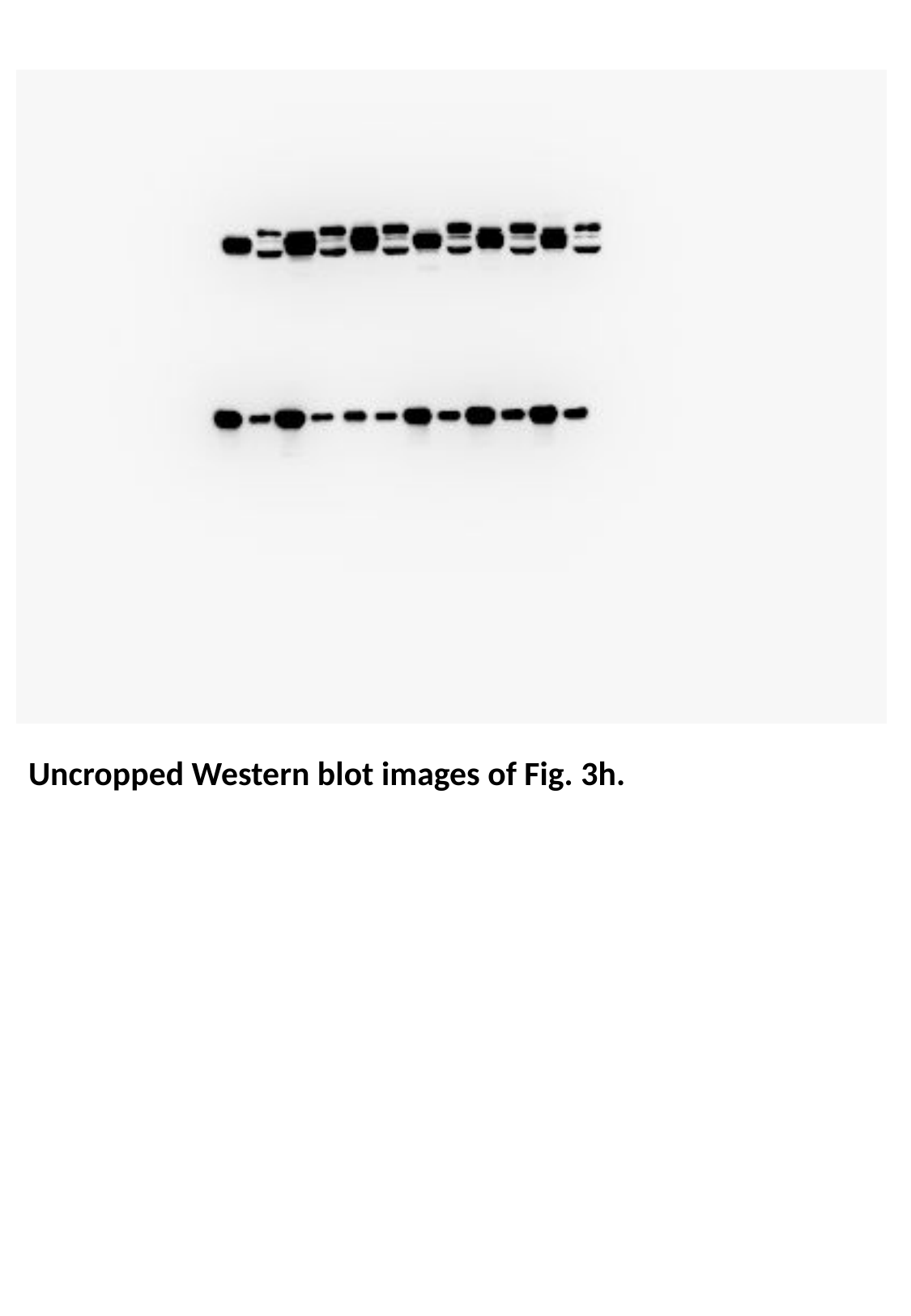

Uncropped Western blot images of Fig. 3h.

## Slide 3
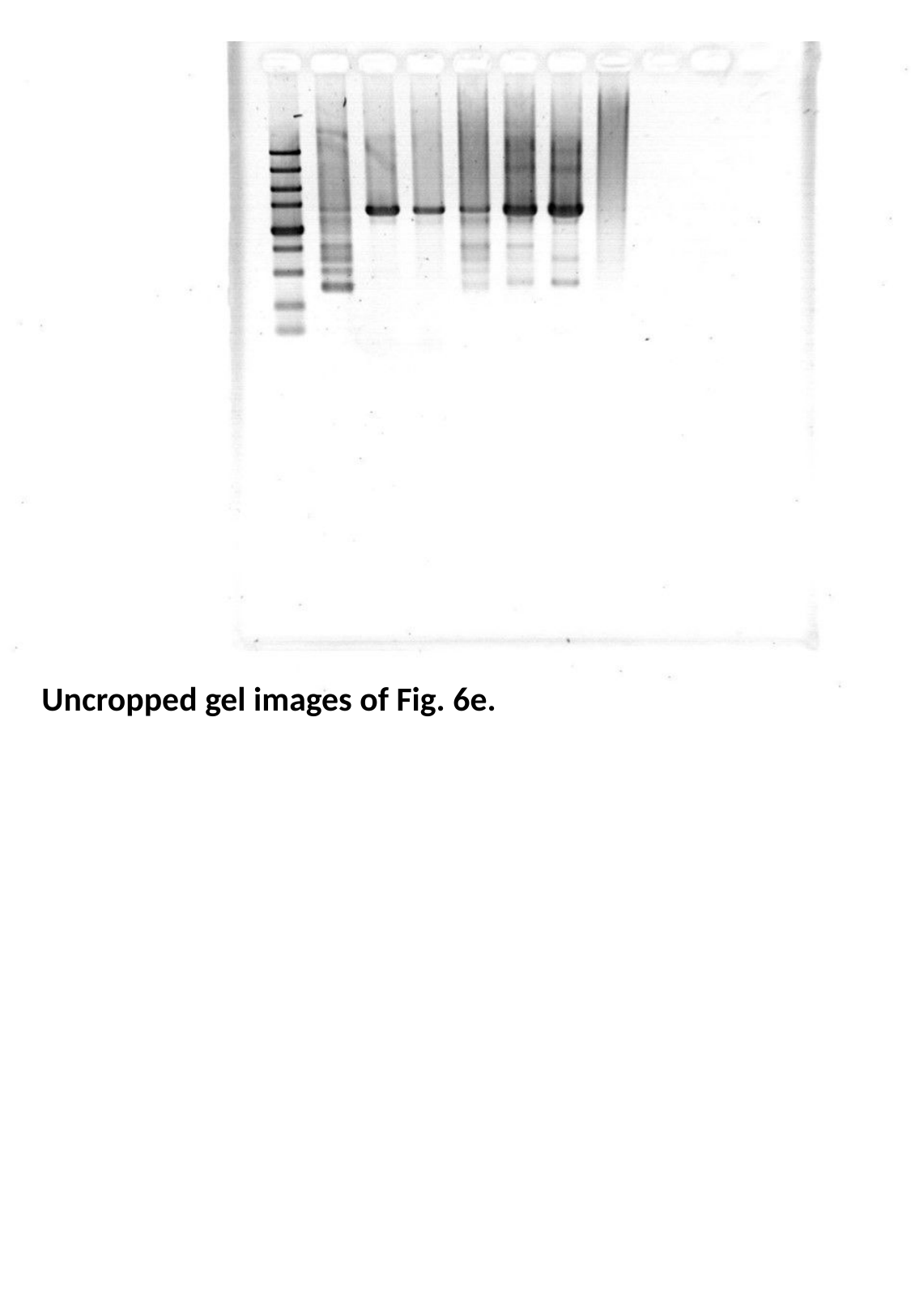

Uncropped gel images of Fig. 6e.

## Slide 4
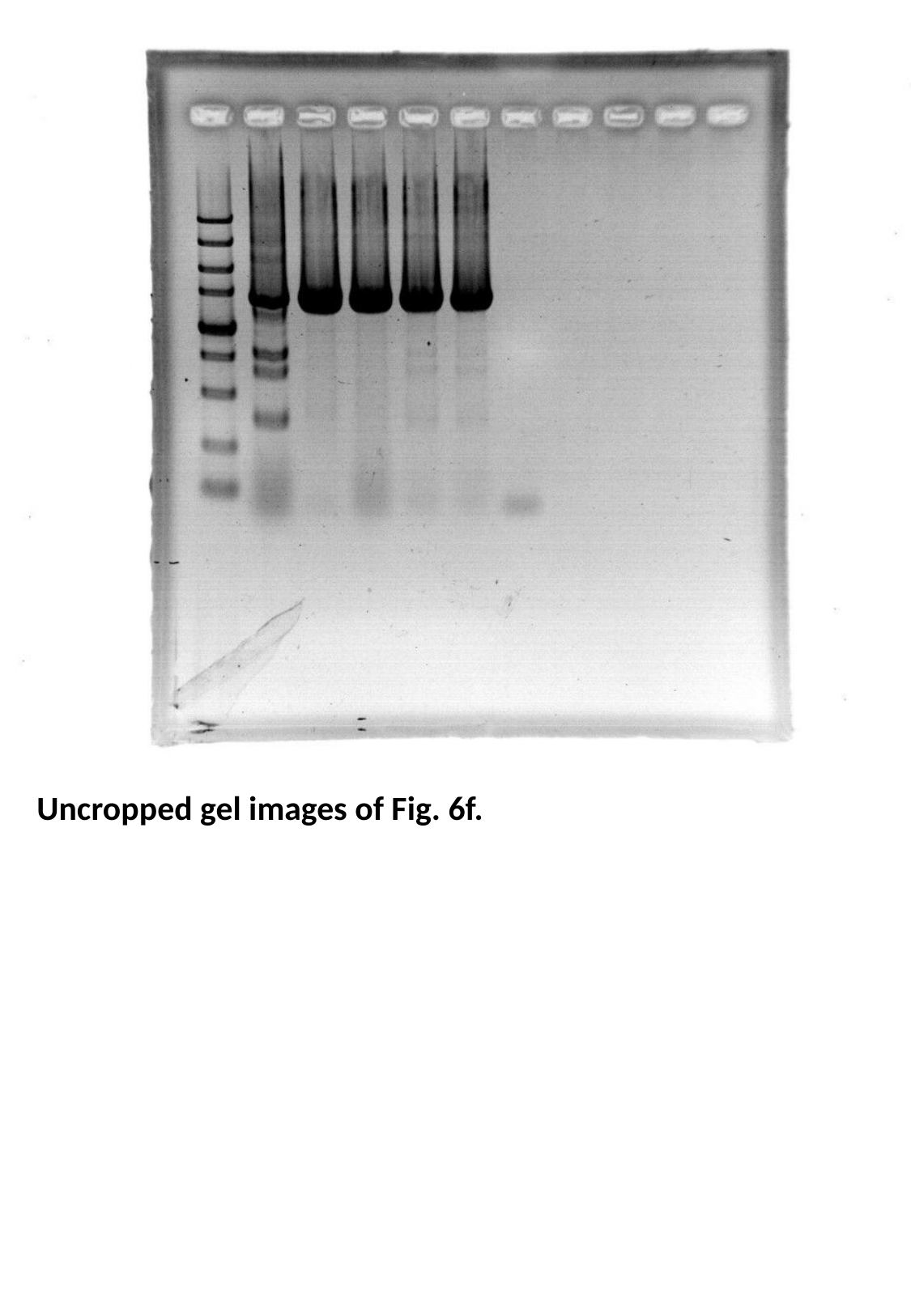

Uncropped gel images of Fig. 6f.

## Slide 5
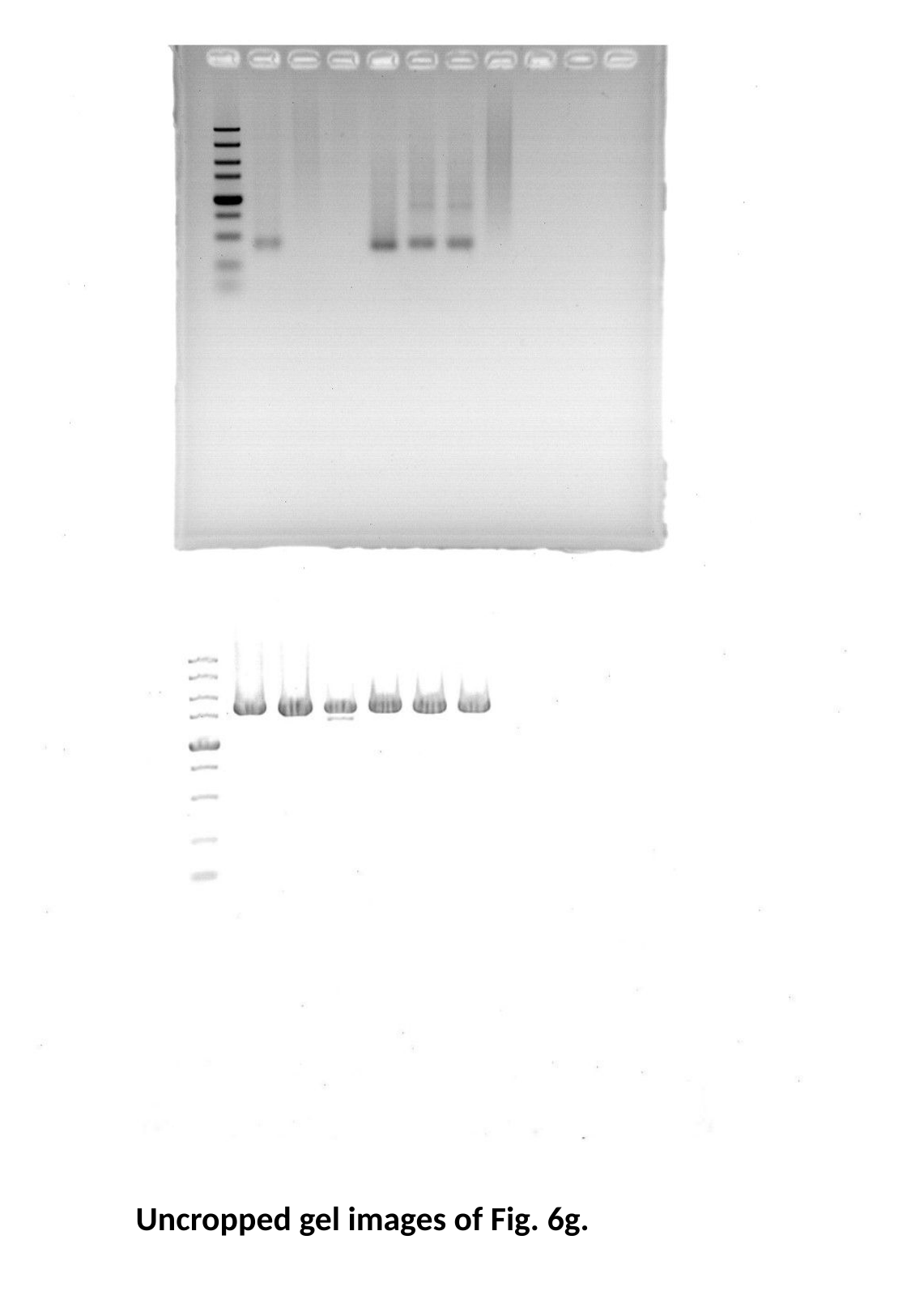

Uncropped gel images of Fig. 6g.

## Slide 6
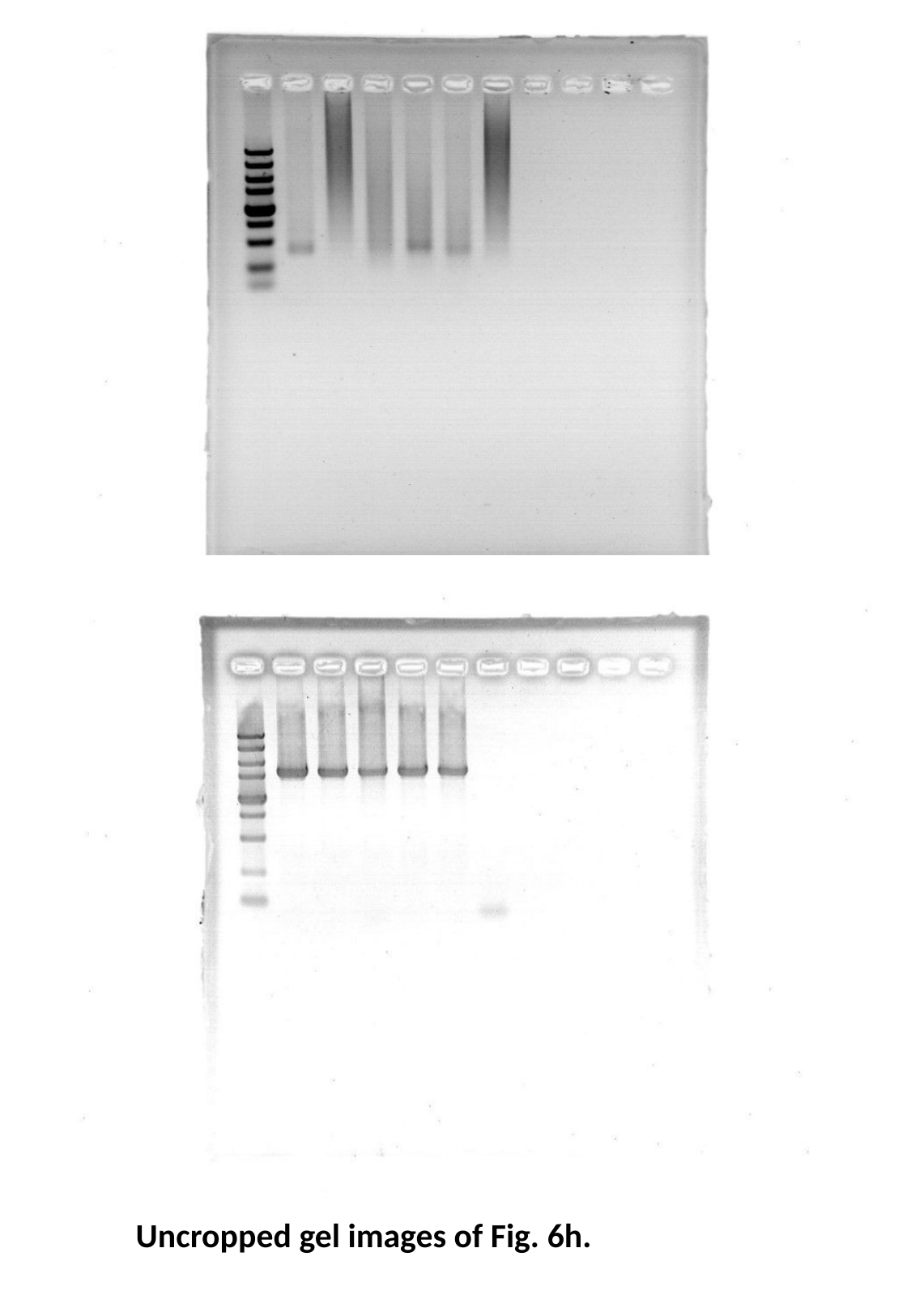

Uncropped gel images of Fig. 6h.

## Slide 7
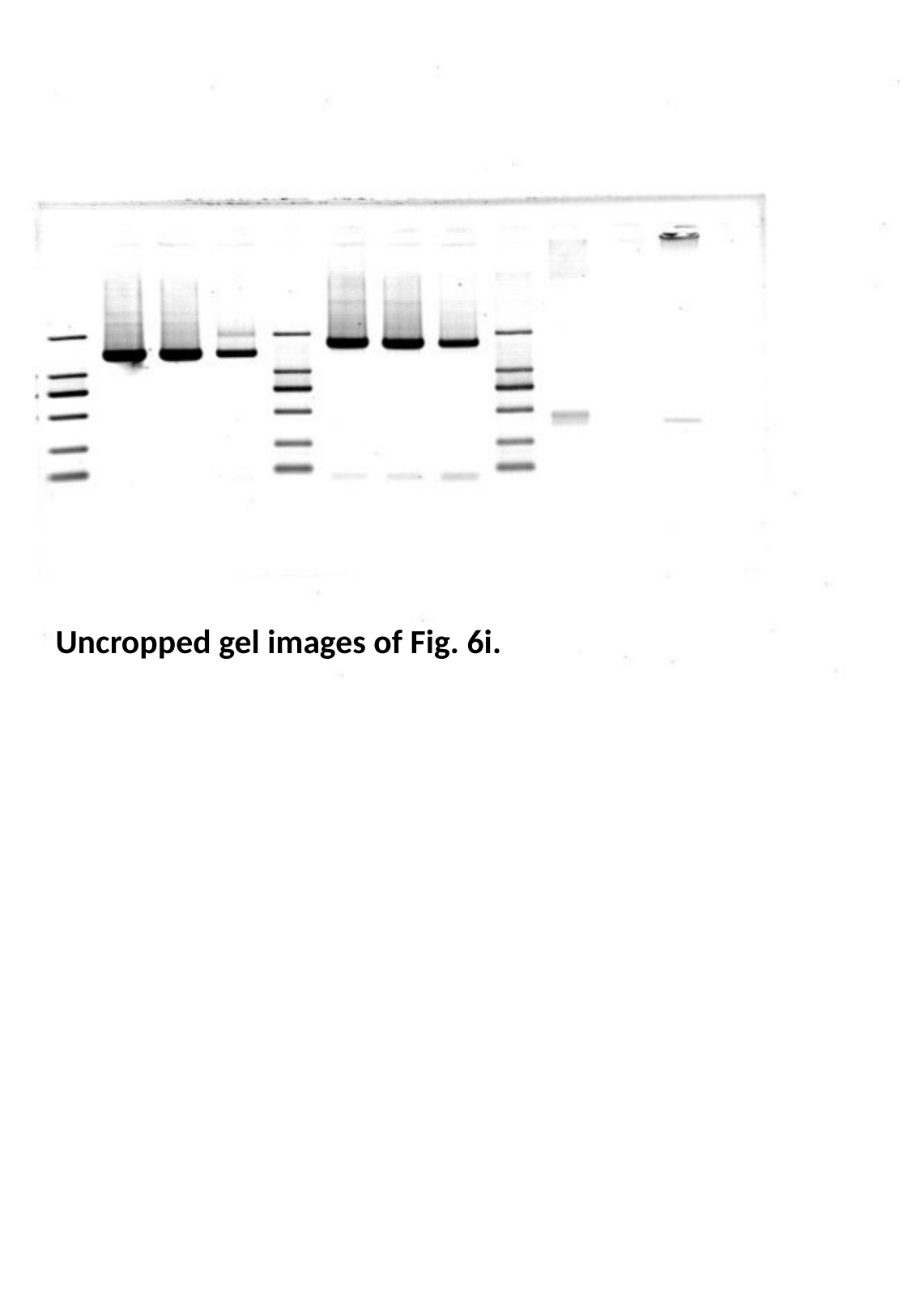

Uncropped gel images of Fig. 6i.
